# Metagenome-assembled genomes from enrichment cultures grown on xenobiotic solvents

**DOI:** 10.1128/mra.00107-24

**Published:** 2024-04-23

**Authors:** Chongjian Jia, Cuiyu Wu, Yingshi Li, Shuhuan Wang, Jinhuan Liu, Jiying Liu, Haimei Su, Xin Tian, Junhui Li

**Affiliations:** 1 Guangdong Eco-Engineering Polytechnic, Guangzhou, China; 2 College of Natural Resources and Environmental Science, South China Agricultural University, Guangzhou, China; 3 Shantou Yuedong Branch, CAUPD Beijing Planning and Design Consultants Ltd., Shantou, China; 4 Department of Biological Science, Vanderbilt Microbiome Innovation Center, Vanderbilt University, Nashville, Tennessee, USA; Indiana University Bloomington, Bloomington, Indiana, USA

**Keywords:** metagenome-assembled genomes (MAGs), shotgun metagenomics, xenobiotic degraders

## Abstract

Microbes play a significant role in the cleanup of xenobiotic contaminants. Based on metagenomes derived from long-term enrichment cultures grown on xenobiotic solvents, we report 166 metagenome-assembled genomes, of which 137 are predicted to be more than 90% complete. These genomes broaden the representation of xenobiotic degraders.

## ANNOUNCEMENT

Xenobiotic contamination has become a global environmental concern due to the enormous rise in the introduction of xenobiotic pollutants into the environment. Microorganisms are key players in xenobiotic remediation, and fundamental research has demonstrated the capability of microbial pure cultures in degrading a wide range of xenobiotics (1, 2). However, the majority of microorganisms remain unexplored because they are challenging to isolate and cultivate (3). It is therefore essential to investigate functional taxa that are not based on isolation. Here, using two hypersaline/saline lake sediments and four contaminated soils, we enriched xenobiotics-degrading consortia grown in serum bottles filled with mineral salt medium supplied with mixed or single xenobiotics [i.e., BTEX (benzene, toluene, ethylbenzene, xylenes], perchloroethylene, trichloroethylene, trichloroethane, 1,4-dioxane, and N,N-dimethylformamide] as their sole carbon source at 25°C at 150 rpm aerobically over a 1-year period as described previously (4, 5). We report 166 metagenome-assembled genomes (MAGs).

Totally, 69 microbial cultures were obtained (see enrichment microcosms at https://doi.org/10.6084/m9.figshare.24201612.v4). Genomic DNA was extracted using the MP FastDNA Spin Kit, and libraries were generated with the Illumina Nextera DNA Flex Library Preparation Kit and sequenced on the Illumina NovaSeq 6000 platform, generating 2.2 billion paired-end reads, averaging 31.8 million reads per sample.

Low-quality sequences were trimmed using TrimGalore v0.6.6 (--paired) (6), and then human sequences were filtered out by aligning to the hg38 human reference genome using Bowtie2 (7). The quality-filtered metagenomes from the same sampling source were both co-assembled and individually assembled using Megahit v1.1.3 (--k-list 21,41,61,81,99; --min-contig-len 500; --min-count 3) (8), and the resulting contigs were then binned using CONCOCT (9), MaxBin2 (10), and metaBAT2 (11), and refined using the metaWRAP (12). Subsequently, the generated bins were dereplicated using dRep v3.4.3 (13) with default parameters. Genome quality was assessed with CheckM v1.2.2 (14), and taxonomic annotation was conducted using GTDB-Tk v2.3.2 (15) based on the database R214.1 (16). The tree was constructed using the *de_novo*_wf workflow in GTDB-Tk and FastTree (17) with WAG + GAMMA models based on an alignment of 120 bacterial single-copy marker genes from the MAGs and visualized with iTOL v6 (18).

**Fig 1:**
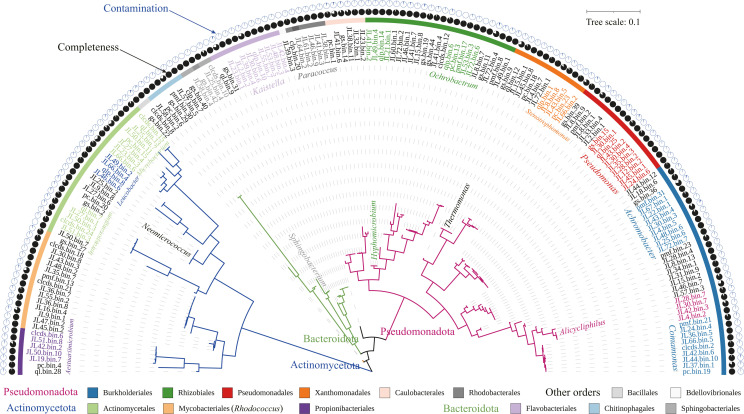
Phylogeny, genome completeness, and contamination of MAGs. Colored branch indicates phylum; colored strip indicates order; italicized taxonomic name indicates genus. See details of MAGs at https://doi.org/10.6084/m9.figshare.24201612.v4.

We recovered 137 high-quality MAGs with >90% completeness and <5% contamination and 29 medium-quality MAGs with >70% completeness and <5% contamination (Fig. 1). The MAGs were estimated to have an average of 94.9% (70%–100%) completeness, 1.1% (0%–4.8%) contamination, GC content of 0.617 (0.376–0.714), and N50 of 84.8  kb (2.2–2929.5 kb). These MAGs were from the phyla *Pseudomonadota* (96), *Actinomycetota* (45), *Bacteroidota* (22), *Bacillota* (2), and *Bdellovibrionota* (1), and the most common bacterial orders were Burkholderiales (33), Actinomycetales (23), Rhizobiales (23), Pseudomonadales (16), Mycobacteriales (15), Xanthomonadales (12), Flavobacteriales (11), Propionibacteriales (7), Caulobacterales (6), Chitinophagales (6), Rhodobacterales (6), and Sphingobacteriales (5). These assembled genomes will expand the representation of xenobiotic degraders, particularly from hypersaline deposits, and provide insights into metabolic capabilities toward xenobiotics.

## Data Availability

The metagenomic data and MAGs analyzed in this study are available in NCBI under the BioProject accession number PRJNA660264.
